# Haloperidol augmentation of fluvoxamine in skin picking disorder: a case report

**DOI:** 10.1186/1752-1947-6-219

**Published:** 2012-07-26

**Authors:** Maria Luca, Costanza Vecchio, Antonina Luca, Carmela Calandra

**Affiliations:** 1 Department of Medical and Surgery Specialties, Psychiatry Unit of the University Hospital ‘Policlinico-Vittorio Emanuele’ of Catania (Sicily), Via S. Sofia 78, Catania, 95100, Italy; 2 Department of Neuroscience of the University Hospital ‘Policlinico-Vittorio Emanuele’ of Catania (Sicily), Via S. Sofia 78, Catania, 95100, Italy

**Keywords:** Fluvoxamine, Haloperidol, Pharmacological association, Skin picking disorder, Treatment

## Abstract

**Introduction:**

Compulsive skin picking, being part of the broader category of impulse control disorders, is considered a residual diagnosis in the *Diagnostic and Statistical Manual of Mental Disorders*, Fourth Edition, Text Revision. It is characterized by excessive scratching or picking of normal skin, or skin with minor surface irregularities, and occurs in 2% of patients attending dermatology clinics. Despite the clinical relevance of this disorder, no clear guidelines are available yet; clinical management is, therefore, compromised and the day-to-day clinical practice is burdened by difficulties. Studies on selective serotonin reuptake inhibitors and anti-epileptic drugs have provided limited results. The association between anti-depressants and anti-epileptics has been found to be beneficial in some impulse control disorders, but in skin picking no previous studies have been conducted on this pharmacological approach. There are very few reports on the efficacy of anti-psychotics in skin picking.

**Case presentation:**

The therapeutic path described in this case report produced good results for a 59-year-old Caucasian woman. The first therapeutic approach, with fluvoxamine and oxcarbazepine was partially effective; then, the suspension of oxcarbazepine and haloperidol augmentation of fluvoxamine were adopted. After 10 weeks, a significant improvement of the disease was observed: the clinical picture and the associated symptoms were nearly solved.

**Conclusions:**

To the best of our knowledge, this is the first article reporting the association of fluvoxamine and haloperidol in skin picking disorder. It might be useful to perform further research regarding the treatment of skin picking disorder: in clinical practice, several variables might limit the choice of certain drugs. Therefore, it would be useful for the clinician to be aware of other therapeutic options.

## Introduction

Compulsive skin picking (also known as neurotic excoriation) is characterized by excessive scratching or picking of normal skin or skin with minor surface irregularities, and it predominantly affects women in their teens to late 30s [1]; although skin picking should deserve more attention, being part of the broader category of impulse control disorders (ICDs), it is considered a residual diagnosis in the *Diagnostic and Statistical Manual of Mental Disorders*, Fourth Edition, Text Revision (DSM-IV-TR) [2]. Regardless, skin picking behaviors are quite frequent in the general population, as demonstrated in a study conducted in the USA which took into consideration conditions ranging from mild to clinically significant ones [3]. In addition, skin picking occurs in 2% of patients attending dermatology clinics and it is associated with medical complications (for example, infection), significant distress and functional impairment [1].

Despite the significant clinical impact of skin picking disorder, no clear therapeutic guidelines are available to date. Different selective serotonin reuptake inhibitors (SSRIs) have been found to be effective in pathologic skin picking, with a significant improvement in picking behavior and a resultant reduction of the lesions [4,5]. However, some reports on SSRIs have provided conflicting results [6].

Further studies have demonstrated the efficacy of anti-epileptic drugs in skin picking disorder [7]. Previous reports suggest that the association between anti-depressants and anti-epileptic drugs might be beneficial in some ICDs [8], but no previous studies seem to have been specifically conducted regarding this pharmacological approach in skin picking.

Only a few reports point out the beneficial role of low doses of neuroleptic drugs for the treatment of skin picking [9,10].

This limited data highlights the need for further research on the therapeutic options to be adopted for this complex disorder. Here, we describe the association of fluvoxamine and haloperidol as a possible therapeutic approach to skin picking disorder. The potential usefulness of this treatment is illustrated in our patient’s case, as described below.

## Case presentation

A 59-year-old Caucasian woman with a three-year history of several dermatological consultations and investigations (results all negative) presented to our Psychiatry Unit for consultation on the advice of her general practitioner and her dermatologist. She had been treated, without success, with various oral and topic antibiotics, even in association with corticosteroids.

The anamnestic data collection revealed that our patient was born with congenital cataracts in both eyes that made her severely sight impaired. Notwithstanding, she is a very active and relatively independent person.

In addition, she experienced traumas in her early life: her three-year-old brother died prematurely, and her 10-year-old sister was burnt to death, in her presence, in a tragic domestic accident.

She began to pick at her skin three years ago, causing herself lesions around the margin of the lips, on her cheeks, chin and nose. Her skin picking was frequent and lasted for hours; it was associated with emotional stress followed by a feeling of relief after picking.

In that period, she was forced to change the arrangement of the furniture and objects in the house due to urgent refurbishment. Being visually impaired, these problems caused her further distress. She reported that her picking behavior was due to different stimuli, such as itching, pain, and burning sensations. She was aware that these feelings, which resulted in compulsive skin picking, were irrational; she did not feel she had any face defects or deformity, but she could not stop picking.

Our patient reported a strong ‘urge’ to pick her skin: a sensation which occurred many times during the day followed by a feeling of relief the moment she picked her skin; all her attempts to resist such an urge required a huge effort and caused serious distress.

This woman has experienced early trauma, stressful events, solitude (being single and living alone), a severe handicap, and she has to take care of herself. She manifested her distress, together with anger and frustration, through her body in a self-destructive way. She picked at her skin causing herself severe lesions; her picking behavior was frequent and sometimes lasted hours.

At her first visit, the general physical examination was within normal limits, apart from the visual impairment and the skin lesions. A dermatological examination revealed the presence of multiple symmetrical facial ulcers and hyper-pigmented areas on normal skin or on pimples, particularly in the area of her cheeks, chin, nose and around her lips. The lesions ranged from a size of around 1cm to 3cm, and appeared in different developmental stages, ranging from ulcers to bruises, scabs, and scar areas, some of which were infected. Our patient, owing to her visual impairment, could not clearly see the injuries that she caused herself. Hence, when shown her magnified photographs, she was shocked by the extent of her facial lesions, which made her wonder what reactions her aesthetic condition would provoke in the people she met.

The results of a mental status examination did not reveal any mood or psychotic disorders, or other Axis I disorders.

The Structured Clinical Interview for DSM-IV Axis II Personality Disorders (SCID-II) [11] revealed traits of obsessive-compulsive personality disorder, as is often observed in skin pickers [12].

Our patient was treated with fluvoxamine 300mg/day and oxcarbazepine 600mg/day, with a reduction of urges to pick her skin and a certain improvement in the extent of the lesions, but she was never completely free from the picking behavior; in addition, she noted considerable drowsiness.

Therefore, we changed the therapeutic program: we suspended oxcarbazepine and we added haloperidol 2mg/ml, seven drops per day (0.7mg/day), to the maintenance therapy of fluvoxamine. After 10 weeks, she came for follow-up, very grateful of what she defined as a ‘full recovery’, having never felt, until now, such a relief from her symptoms. With the new therapy, unlike the previous one, she was able to completely master her tendency to pick her skin, with the subsequent significant improvement of her clinical conditions.

## Discussion

‘Nervous habits’ such as nail biting, hair pulling and skin picking are often seen by general practitioners as common and benign, despite the disfiguring effects and the distress related to these disorders [3]. Pickers, in particular, experience a variety of psychosocial difficulties, including social embarrassment and avoidance, loss of productivity at work, anxiety, depression and suicidal ideation [13]. Even if pathological skin picking is quite a frequent and disabling disorder, it is not classified as a separate disorder in the DSM-IV-TR. Some authors have suggested skin picking to be a part of the obsessive-compulsive spectrum disorders; as a result, the DSM-V will probably include it in this diagnostic group as a better-defined diagnostic label (http://www.dsm5.org). Skin picking is thus achieving a certain level of attention; however, at present research for the treatment of this disorder is sparse and no clear guidelines are available. Consequently, the choice of the therapy is almost completely left in the hands of the clinicians.

In our patient’s case, our therapeutic choice initially consisted of oxcarbazepine and fluvoxamine; following a partial success, our patient presented an almost complete recovery after we suspended oxcarbazepine and added haloperidol. In the literature, a limited number of controlled trials have suggested the efficacy and tolerability of SSRIs in the treatment of skin picking; this is one of the reasons this disorder has been related with obsessive-compulsive disorder. In fact, these anti-depressants have been extensively and successfully tested in obsessive-compulsive disorder and, moreover, skin picking is frequently associated with depression [4,5]. However, there are some reports that these drugs could even trigger a skin picking behavior [6]. The association between anti-depressant and anti-epileptic drugs has been found to be beneficial in some ICDs [8], but to date no data about this pharmacological approach in skin picking are available. With regard to anti-psychotics, olanzapine and paliperidone have been considered efficacious in only a few cases of skin picking [9,10]. In our patient’s case we preferred to use haloperidol in place of olanzapine because of the risk of metabolic side effects, especially, in overweight individuals, as was the situation with our patient. Paliperidone was not used because drug-induced obsessive symptoms have been reported with this substance [14] and this effect might make its use risky in a disease such as skin picking. The efficacy of haloperidol was probably due to its anti-dopaminergic function. Some authors believe self-injurious behaviors may be the result of an excessive stimulation of dopamine receptors in mesolimbic structures [15].

In this context of growing knowledge, the ‘future’ of skin picking, a very complex but fascinating disorder, has yet to be written; further research on the potential usefulness of other drugs is needed, also taking into account new findings in the neurobiological field.

## Conclusions

The clinical impact of skin picking disorder should not be underestimated, for the distress that characterizes it and for its disfiguring effects, often on exposed parts of the body. The choice of the right therapy is a challenge in clinical practice, since several variables might limit the use of certain drugs. To the best of our knowledge, this is the first case reporting the efficacy of the association of haloperidol and fluvoxamine in this disorder. It would be useful to improve research about skin picking and its treatment, to provide clinicians a certain, widely recognized, range of therapeutic options.

## Consent

Written informed consent was obtained from the patient for publication of this case report and any accompanying images. A copy of the written consent is available for review by the Editor-in-chief of this journal.
